# ACBE, a new base editor for simultaneous C-to-T and A-to-G substitutions in mammalian systems

**DOI:** 10.1186/s12915-020-00866-5

**Published:** 2020-09-23

**Authors:** Jingke Xie, Xingyun Huang, Xia Wang, Shixue Gou, Yanhui Liang, Fangbing Chen, Nan Li, Zhen Ouyang, Quanjun Zhang, Weikai Ge, Qin Jin, Hui Shi, Zhenpeng Zhuang, Xiaozhu Zhao, Meng Lian, Jiaowei Wang, Yinghua Ye, Longquan Quan, Han Wu, Kepin Wang, Liangxue Lai

**Affiliations:** 1 CAS Key Laboratory of Regenerative Biology, Guangdong Provincial Key Laboratory of Stem Cell and Regenerative Medicine, Guangzhou Institutes of Biomedicine and Health, Chinese Academy of Sciences, Guangzhou, 510530 China; 2 Research Unit of Generation of Large Animal Disease Models, Chinese Academy of Medical Sciences (2019RU015), Guangzhou, 510530 China; 3 University of Chinese Academy of Sciences, Beijing, 100049 China; 4 Bioland Laboratory (Guangzhou Regenerative Medicine and Health Guangdong Laboratory, GRMH-GDL), Guangzhou, 510005 China; 5 Institute of Physical Science and Information Technology, Anhui University, Hefei, 230601 China

**Keywords:** Adenine and cytosine base editor (ACBE), Simultaneous C-to-T and A-to-G conversions, Mammalian systems

## Abstract

**Background:**

Many favorable traits of crops and livestock and human genetic diseases arise from multiple single nucleotide polymorphisms or multiple point mutations with heterogeneous base substitutions at the same locus. Current cytosine or adenine base editors can only accomplish C-to-T (G-to-A) or A-to-G (T-to-C) substitutions in the windows of target genomic sites of organisms; therefore, there is a need to develop base editors that can simultaneously achieve C-to-T and A-to-G substitutions at the targeting site.

**Results:**

In this study, a novel fusion adenine and cytosine base editor (ACBE) was generated by fusing a heterodimer of TadA (ecTadA^WT/*^) and an activation-induced cytidine deaminase (AID) to the N- and C-terminals of Cas9 nickase (nCas9), respectively. ACBE could simultaneously induce C-to-T and A-to-G base editing at the same target site, which were verified in HEK293-EGFP reporter cell line and 45 endogenous gene loci of HEK293 cells. Moreover, the ACBE could accomplish simultaneous point mutations of C-to-T and A-to-G in primary somatic cells (mouse embryonic fibroblasts and porcine fetal fibroblasts) in an applicable efficiency. Furthermore, the spacer length of sgRNA and the length of linker could influence the dual base editing activity, which provided a direction to optimize the ACBE system.

**Conclusion:**

The newly developed ACBE would expand base editor toolkits and should promote the generation of animals and the gene therapy of genetic diseases with heterogeneous point mutations.

## Background

The rapid development of gene editing technologies (ZFNs, TALENs, and CRISPR/Cas9) plays an increasingly important role in biomedical and agricultural fields [1]. Many desirable agricultural traits of crops and livestock and human genetic diseases arise from multiple single nucleotide polymorphisms (SNPs) or multiple point mutations with heterogeneous base substitutions at the same locus [2–7]. Therefore, base editing of a genome in multiple sites with heterogeneous base substitutions is necessary to achieve favorable traits in agriculture, establish human disease animal models, and treat human hereditary diseases [8, 9]. Genome editing with either the CRISPR-based cytosine base editor (CBE) or the adenine base editor (ABE) can be used for C-to-T or A-to-G base substitutions in a high efficiency, but both editors are not applicable for correction of other variants such as base transconversion, small insertions, and deletions (indels) [10–12]. Compared with base editors, the prime editing system (PE), a “search-and replace” genome editing tool, can induce base substitutions in more extended regions [13]. However, the efficiency of PE for making transition point mutations was reported much lower than that of both CBE and ABE, which makes PE difficult to be used to generate organisms or correct genetic diseases that need base editing of a genome in multiple sites with heterogeneous base substitutions [13–15].

In an earlier report, Li et al. fused APOBEC3A-ecTadA-ecTadA7.10 or ecTadA-ecTadA7.10-APOBEC3A, to the N terminus of nCas9 (D10A), together with UGI at the C terminus of nCas9 (D10A), and successfully induced C-to-T and A-to-G conversions simultaneously at the same target site in plants [16]. A preprint (recently published in *Nature Biotechnology*) presents a novel base editor, Target-ACE, which integrates the abilities of ABEs and CBEs and can simultaneously induce C-to-T and A-to-G base editing in a mammalian immortalized cell line, human embryonic kidney HEK293Ta cells [17]. However, Target-ACE was not verified in primary somatic cells and other mammalian cells, which is vital for applying the base editor for generation of animals through the somatic cell nuclear transfer approach and correction of human genetic diseases. A version of CBEs [11], namely, Target-AID, has two unique features: one is that the main editing window of protospacer positions is within 1–5 instead of 4–8 as that in other APOBEC1 based CBEs and the second is that PmCDA1, the cytidine deaminase used in this system, is fused to the C terminus of Cas9 nickase (nCas9) instead of the N terminus. By contrast, A•T base pair to G•C base pair conversions in an ABE system are performed within the editing window of protospacer positions 4–8, and adenine deaminase, a heterodimer of TadA (ecTadA^WT/*^), is fused to the N terminus of nCas9 [12]. The differences of the position of deaminases and the editing windows of deaminases between target-AID and ABE make them complementary with each other in terms of structure. Therefore, in this study, we fused Target-AID and ABE7.10 to generate a new base editing tool, namely, ACBE. We verified that ACBE could simultaneously generate C-to-T (G-to-A) and A-to-G (T-to-C) not only in the target sites of an immortalized cell line (HEK293 cells), but also in primary somatic cells such as mouse embryonic fibroblasts (MEFs) and porcine fetal fibroblasts (PFFs). Moreover, we confirmed that the efficiency of simultaneous C-to-T and A-to-G conversions could be improved through optimization of linker length and sgRNA spacer length. Dual-function ACBE would expand the toolkit of base editors and has the potential biomedical and agricultural applications.

## Results

### Evaluation of the function of ACBE in a HEK293-EGFP cell line

**Fig. 1:**
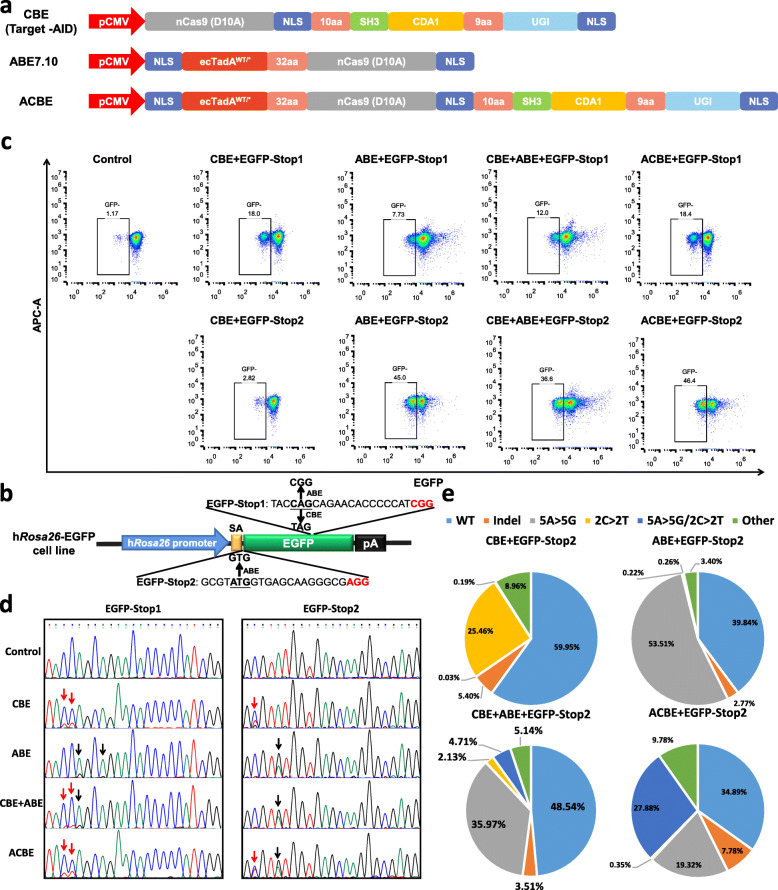
Simultaneous C-to-T and A-to-G base substitutions induced by the ACBE system. **a** Architectures of the dual-function base editing system, ACBE, and individual base editing systems, namely Target-AID and ABE7.10. ecTadA^W^^T^^/^^*^, a heterodimer of an evolved *Escherichia coli* TadA and a wild-type TadA; aa, amino acid; NLS, nuclear localization signal; UGI, inhibitor of uracil DNA glycosylase. **b** Schematic of the HEK293-EGFP reporter cell line and two targeting sgRNA (EGFP-Stop1 and EGFP-Stop2), which could induce a stop codon and mutation of start codon, respectively. **c** The representative results of flow cytometry showed the proportion of EGFP-negative cells edited by different base editors with the corresponding sgRNA. Three independent experiments were performed. **d** The representative Sanger sequencing results of the cell samples transfected with different base editors showed the target base substitutions in the targeting sites EGFP-Stop1 and EGFP-Stop2. The C-to-T and A-to-G substitutions were marked with red and black arrows, respectively. **e** The editing effects on the target site EGFP-Stop2 generated by the ACBE and CBE + ABE system. Different parts with various colors in the pie chart showed the frequencies of the corresponding mutation type

The PmCDA1 and UGI of Target-AID were fused to the C terminus of ABE-7.10, an optimized version of the ABE system, with a nuclear localization signal (NLS) located at the N and C terminals to engineer a new base editor with the abilities of Target-AID and ABE-7.10 (Fig. 1a). To verify whether the size of base editor expression cassettes (CBE, ABE, and ACBE) influence the expression levels of base editors, additionally, base editor expressing vectors fused to the base editor with enhanced green fluorescent protein (EGFP) by P2A were constructed (Additional file 1: Fig. S1a). The HEK293 cells were transfected with Plasmids CBE-EGFP (7.2 μg), ABE-EGFP (7.2 μg), and ACBE-EGFP (8 μg), respectively. Two-day post transfection, the cells were collected and analyzed by flow cytometry. The results showed that the proportion of EGFP-positive cells (52.1%, 52.0%, and 55.7%) and the EGFP intensity had no significant differences among the three groups, indicating that the size of base editors did not influence the expression levels (Additional file 1: Fig. S1b-d).

Next, we demonstrated whether ACBE could simultaneously generate C-to-T and A-to-G conversions in the same target site. An HEK293-EGFP cell line expressing an EGFP, which is under the control of an endogenous *hRosa26* promoter, was used to test the dual base editing function of ACBE [18]. Two EGFP-targeting sgRNAs, namely, EGFP-Stop1 and EGFP-Stop2, which corresponded to Target-AID and ABE-7.10, respectively, were designed and constructed (Fig. 1b). Generation of a premature stop codon and disruption of the start codon in the EGFP gene would be performed by C-to-T conversion (EGFP-Stop1) and A-to-G substitution (EGFP-Stop2), respectively, and both point mutations were expected to block the EGFP expression of HEK293-EGFP cells. The ACBE vector and their corresponding sgRNAs were co-electroporated into HEK293-EGFP cells. The transfection of Target-AID (referred to as CBE in figures) or ABE7.10 (referred to as ABE in figures) was used as a negative control. In addition, the previous report showed that ABE combined with CBE could introduce A-to-G and C-to-T substitutions simultaneously in mouse embryos [19]. Target-AID plus ABE7.10 (referred to as CBE + ABE in figures) were used as a positive control. The cells were collected 5 days after transfection and flow cytometry assay was performed to detect the proportion of the EGFP-negative cells. As shown in Fig. 1c and Additional file 1: Fig. S2a, the EGFP expression was silent in 18.4% of the cells when the ACBE was transfected with EGFP-Stop1, and this proportion was almost equal to that resulted from CBE (18.0%) and CBE + ABE (12.0%). Moreover, 7.73% EGFP-positive cells were observed in bulks of HEK293-EGFP cells transfected with ABE and EGFP-Stop1 probably because EGFP-Stop1 and ABE-mediated missense point mutation (Q184R) shifted the wavelength of excitation light or the intensity of green fluorescence [20]. When ACBE was transfected with EGFP-Stop2, the EGFP expression was silent in 42.5% of the cells, similar to that resulted from ABE (45.0%) and CBE + ABE (36.6%). These results indicated that the ACBE system independently maintained CBE and ABE functions in human cells. The collected cell samples were further subjected to Sanger sequencing. The results showed that simultaneous C-to-T substitution at position 2 and A-to-G substitution at position 5 were observed in the ACBE-transfected HEK293-EGFP cells at the EGFP-Stop2 target site, but not in the CBE + ABE transfected HEK293-EGFP cells. Although simultaneous C-to-T substitutions at positions 3 and 4, and A-to-G substitution at position 5 occurred in the CBE + ABE transfected HEK293-EGFP cells at the EGFP-Stop1 target site, only C-to-T substitutions at positions 3 and 4 were found in the ACBE-transfected cells (Fig. 1d and Additional file 1: Fig. S2b, 2c). Whether these simultaneous substitutions of these two loci occurred in the same DNA strand, polymerase chain reaction (PCR) products were analyzed by TA-cloning sequencing. The Sanger sequencing results of TA-cloning confirmed that only the cell samples co-transfected EGFP-Stop2 with ACBE showed simultaneous C-to-T and A-to-G substitutions at the same DNA allele (Additional file 1: Fig. S2d). Amplicon deep sequencing results further confirmed that the efficiency of simultaneous C-to-T and A-to-G substitutions at the same DNA allele was 27.88% for ACBE at the EGFP-Stop2 target site, but significantly higher than that with the CBE + ABE (4.71%) (Fig. 1e). Furthermore, the simultaneous C-to-T and A-to-G substitutions at the same DNA strand were almost not observed in cells transfected with either individual CBE (0.19%) or ABE (0.22%) (Additional file 1: Fig. S2e). These results indicated that the ACBE system could induce simultaneous C-to-T and A-to-G substitutions at the same target locus and create the heterologous mutation at the same DNA strand with high efficiency with single sgRNA.

### ACBE-mediated simultaneous C-to-T and A-to-G conversions in the endogenous loci of HEK293 cells

**Fig. 2:**
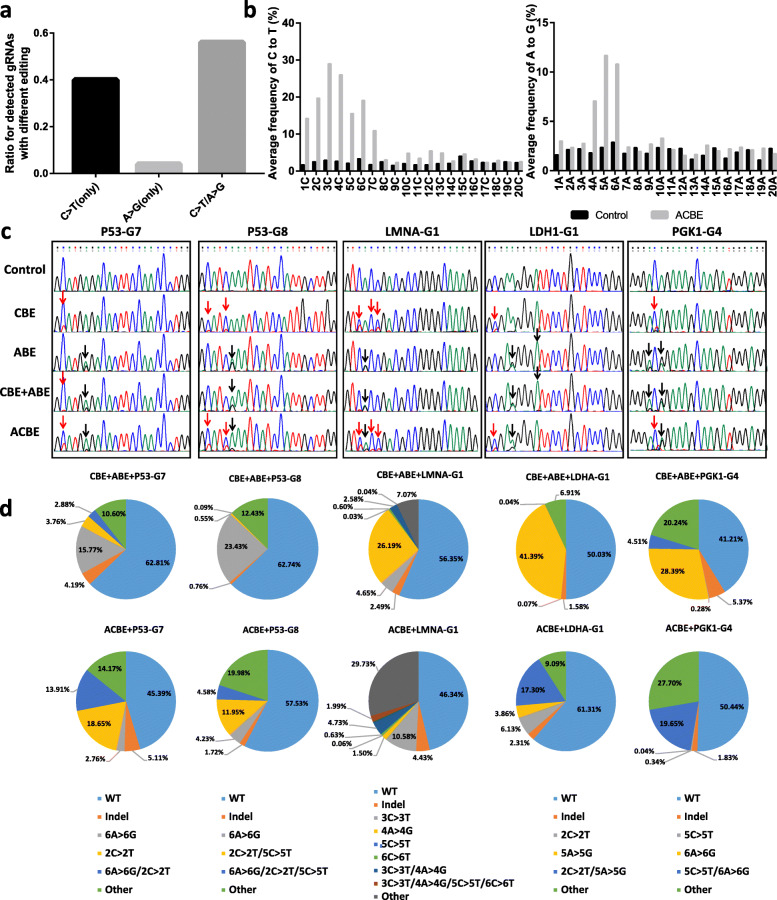
Base editing of ACBE in endogenous loci of HEK293 cells. **a** Summary of base editing type with ACBE at 45 sgRNAs targeting the human endogenous loci. **b** Average C-to-T and A-to-G base editing spectrum of ACBE for endogenous targeting sites in HEK293 cells based on the statistical analysis of the 45 sgRNAs. The values indicated the mean, and the untreated samples served as control. **c** The representative Sanger sequencing results of the cell samples transfected with different base editors showed the target base substitutions in the targeting sites P53-G7, P53-G8, LMNA-G1, LDHA-G1, and PGK1-G4. The C-to-T and A-to-G substitutions were marked with red and black arrows, respectively. Three independent experiments were performed. **d** The editing effects on the target sites P53-G7, P53-G8, LMNA-G1, LDHA-G1, and PGK1-G4 were generated by ACBE and CBE + ABE, respectively. Different parts with various colors in the pie chart showed the frequencies of the corresponding mutation type. The result was based on the representative amplicon deep sequencing (*n* = 3)

Forty-five sgRNAs targeting seven human endogenous genes (15 for *TP53*, 4 for *LMNA*, 14 for *PGK1*, 3 for *TDP43*, 3 for *CTNNB1*, 2 for *AAVS1*, and 4 for *LDHA*) were designed and constructed (Additional file 2: Table S1). Forty-three of the sgRNAs covered cytosine and adenine at different positions of editing windows relative to the PAM sites, whereas two of them only had adenines, but not cytosines in their editing window of sgRNA. ACBE and sgRNA expressing vectors were co-transfected into wild-type HEK293 cells. Three-day post transduction, genomic DNAs were extracted and screened to determine whether simultaneous C-to-T and A-to-G conversions occurred at the target sites. PCR products surrounding the target sites were subjected to Sanger sequencing. Base editing frequency was quantified with EditR [21]. The results showed that 25 sgRNAs in the ACBE system could simultaneously achieve C-to-T and A-to-G conversions (Fig. 2a). For the remaining 20 sgRNAs, 18 sgRNAs achieved only significant C-to-T base editing and 2 sgRNAs achieved only A-to-G base substitutions. Of these sgRNAs, 11 sgRNAs had no A in positions 4–6 and 2 sgRNAs had no C in positions 1–7 (Additional file 2: Table S2). The average frequencies of C-to-T and A-to-G for every targeting nucleotide in different positions relative to PAM (referred to PAM as + 21 to + 23 bp) indicated that the region with a high efficiency of C-to-T conversion spanned from + 1 to + 7 bp, ranging from 10.92 to 29.00%, whereas that of A-to-G point mutation was from + 4 to + 6 bp, with efficiencies ranging from 7.07 to 11.68% (Fig. 2b). The analysis of targeting sequences of 45 sgRNAs and their corresponding efficiencies of base editing showed that ACBE performed preference C-to-T conversions in TC motif, whereas no obvious motif preference for A-to-G point mutations (Additional file 1: Fig. S3). These results indicated that the ACBE had a similar spectrum and the editing efficiency in C-to-T conversions to Target-AID [11], whereas the narrowed editing spectrum and the reduced editing efficiency in A-to-G conversions were observed compared with that of ABE7.10 [12].

Five sgRNAs (P53-G7, P53-G8, LMNA-G1, LDHA-G1, and PGK1-G4) targeting four of the human endogenous genes (*TP53*, *LMNA*, *LDHA*, and *PGK1*) were selected to validate the base editing effects of the ACBE system on endogenous genes in detail. The Sanger sequencing results showed that the mutant peak of C-to-T was observed in the cells transfected with CBE and ACBE but not with CBE + ABE **(**Fig. 2c and Additional file 1: Fig. S4). However, only A-to-G conversions were detected in cells transfected with ABE, CBE + ABE, and ACBE. These findings indicated that the ACBE system maintained an efficient dual functionality for C-to-T and A-to-G mutation, consistent with the results of the reporter assay. Conversely, CBE + ABE showed the prior editing of A-to-G and reduced C-to-T editing. The C-to-T base editing efficiency of ACBE was comparable with Target-AID, whereas the A-to-G editing efficiency was reduced compared with that of ABE7.10. Notably, the CBE + ABE group showed a significantly reduced efficiency of C-to-T substitutions and maintained the normal function of A-to-G conversion among the five target sites, indicating that ABE7.10 might have better binding competitiveness than Target-AID to the same target DNA locus. Deep sequencing was conducted to analyze the effect of simultaneous C-to-T and A-to-G substitutions that occurred at a single target site of the same DNA strand by different base editing systems. The results revealed that the ACBE system could efficiently induce simultaneous heterogeneous base editing (A-to-G and C-to-T) at the same DNA strand with efficiencies of 13.91% (2C > 2 T and 6A > 6G) for P53-G7, 4.58% (2C > 2 T, 5C > 5 T, and 6A > 6G) for P53-G8, 4.73% (3C > 3 T and 4A > 4G) and 1.99% (3C > 3 T, 4A > 4G, 5C > 5 T, and 6A > 6G) for LMNA-G1, 17.30% (2C > 2 T and 5A > 5G) for LDHA-G1, and 19.65% (5C > 5 T and 6A > 6G) for PGK1-G4, whereas the CBE + ABE system only had efficiencies of 2.88% for P53-G7, 0.09% for P53-G8, 2.58% and 0.04% for LMNA-G1, 0.04% for LDHA-G1, and 4.51% for PGK1-G4 (Fig. 2d). The individual base editor CBE or ABE almost could not induce simultaneous C-to-T and A-to-G substitution at the same DNA strand with a frequency below 0.23% in the five tested loci **(**Additional file 1: Fig. S5). The abovementioned results showed an advantage for ACBE to induce simultaneous C-to-T and A-to-G mutation at the same DNA strand. Except for heterogeneous base editing, homogeneous base editing (A-to-G or C-to-T) was also observed in the CBE + ABE and ACBE groups **(**Fig. 2d, e). In addition, the proportions of indels formation in the five tested loci by different base editors were analyzed. The indels frequency of ACBE (1.72–5.11%) was not increased compared with CBE (1.45–6.78%), ABE (0.77–4.09%), and CBE + ABE (1.58–5.37%), indicating that ACBE maintained the safe genome editing potential with a low frequency of DNA double-strand breaks (DSB) (Fig. 2d and Additional file 1: Fig. S5).

In addition, the off-target effects of ACBE on the potential off-target sites (POTs) were tested in the HEK293 gene loci. Eight off-target sites for P53-G7 and four off-target sites for P53-G8 were selected to analyze the off-target effects between ACBE and individual base editor (CBE and ABE). From the Sanger sequencing results, no evident off-target effects were observed at POTs for CBE, ABE, and ACBE (Additional file 1: Fig. S6). Moreover, we tested the off-target effects of ACBE and CBE + ABE with LMNA-G1, LDHA-G1, and PGK1-G4. For each sgRNA, four top POTs were analyzed with Sanger sequencing. The results of the tested off-target sites showed no mutation peaks except for the site PGK1-G4-OT1, which showed an evident A-to-G mutation peak induced by CBE + ABE and a mild mutation peak induced by ACBE, indicating that ACBE had better specificity than CBE + ABE with PGK1-G4 sgRNA (Additional file 1: Fig. S7). All the results of the abovementioned off-target confirmed that the ACBE system showed high specificity in base editing, comparable to individual base editors.

### ACBE-mediated simultaneous C-to-T and A-to-G conversions in primary somatic cells

**Fig. 3:**
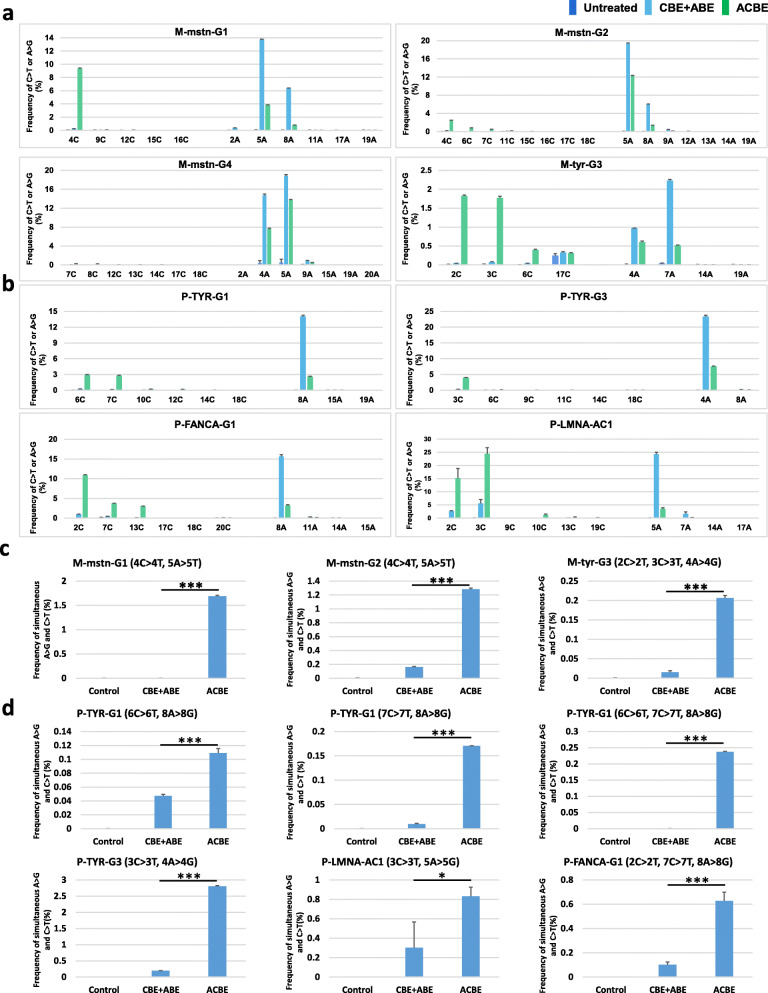
ACBE-mediated dual-function base editing in MEFs and PFFs. **a** The base editing effects of ACBE in *mstn* and *tyr* loci of MEFs; **b** The base editing effects of ACBE in *TYR*, *FANCA*, and *LMNA* loci of PFFs. The efficiencies of base editing (**a**, **b**) were quantified from amplicon deep sequencing. Values and error bars indicate mean ± s.e.m. of three independent experiments. An untreated cell sample served as control. **c**, **d** The efficiencies of ACBE-mediated simultaneous C-to-T and A-to-G base substitutions in the targeting loci of MEFs (**c**) and PFFs (**d**) were calculated the reads of amplicon deep sequencing. N.S., not significant (*P* > 0.05); *(0.05 > *P* ≥ 0.001); **(0.01 > *P* ≥ 0.0001); ***(*P* < 0.0001) (two-tailed *t* test)

**Fig. 4:**
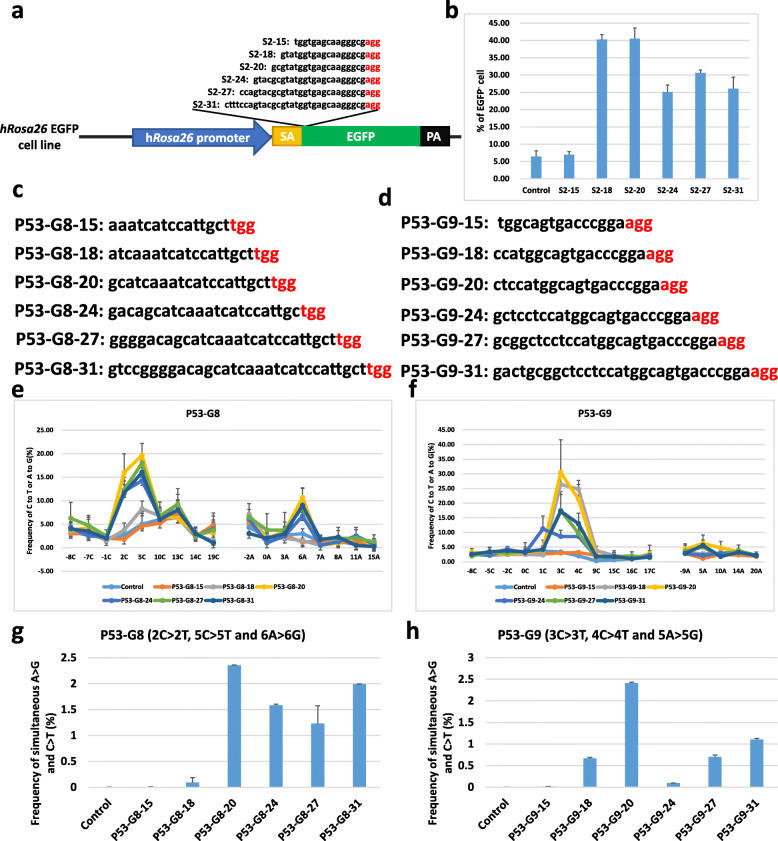
ACBE-mediated base editing effects with different spacer lengths. **a** Schematic design for different spacer length of EGFP-Stop2. The red sequence indicated the PAM. **b** Flow cytometry results for testing different spacer lengths of EGFP-Stop2, *n* = 3. Untreated cell samples served as control. **c**, **d** The sequences represented the serial sgRNAs with different spacer lengths at P53-G8 (**c**) and P53-G9 (**d**) loci. The red sequence indicated the PAM. **e**, **f** Summary of ACBE-mediated base editing patterns and efficiencies of all C and A in P53-G8 (**e**) and P53-G9 (**f**) sites using sgRNAs with different spacer lengths. All values and error bars above indicate mean ± s.e.m. of three independent experiments. The controls were untreated cell samples. **g**, **h** The frequencies of simultaneous A > G and C > T mutation for P53-G8 (**g**) and P53-G9 (**h**) loci with different spacer length gRNA

In previous reports, dual-function base editors, saturated targeted endogenous mutagenesis editors (STEMEs), and Target-ACE, were verified the function of simultaneous C-to-T and A-to-G conversions in plants and HEK293Ta cell line, respectively [16, 17]. Whether simultaneous C-to-T and A-to-G substitutions could be induced in primary somatic cells and other mammalian cells was not validated. Next, the dual-function effects of ACBE were tested in primary somatic cells, including MEFs and PFFs. Four sgRNAs targeting the endogenous genes (3 for *mstn* and 1 for *tyr*) of MEFs and four sgRNAs targeting the endogenous genes (2 for *TYR*, 1 for *FANCA*, and 1 for *LMNA*) of PFFs were designed and constructed (the sgRNA targeting sequences were shown in Additional file 2: Table S1). The individual sgRNA (4 μg) with CBE + ABE (7.2  μg + 7.2  μg) or ACBE (8 μg) expressing vectors was co-transfected into the primary somatic cells. Three days after transfection, the cell samples were collected for amplicon deep sequencing. The read ratios of amplicon deep sequencing were used to represent the efficiencies of base editing **(**Fig. 3a, b). The results showed that ACBE-mediated simultaneous C-to-T and A-to-G point mutations occurred in three tested targeting loci of MEFs (M-mstn-G1, G2, and M-tyr-G3) and four targeting loci of PFFs (P-TYR-G1, G3, P-FANCA-G1, and P-LMNA-AC1). Only a detectable A-to-G point mutation was observed for M-mstn-G4 because only suitable adenines were presented in the editing window of the ACBE system. However, for the CBE + ABE system, simultaneous C-to-T and A-to-G point mutations were only found at the P-LMNA-AC1 targeting site, whereas only A-to-G substitutions occurred at other targeting loci. In addition, these results showed that the ACBE system maintained the prior C-to-T point mutation, whereas the CBE + ABE system showed the prior mutation of A-to-G, which was consistent with the characteristic of base editing in HEK293 **(**Fig. 3a, b**)**. The editing efficiency of C-to-T conversions and A-to-G conversions reached 9.35% and 13.71% for ACBE in the tested loci of MEFs, respectively (Fig. 3a), whereas 24.45% of C-to-T and 7.58% of A-to-G substitutions in PFFs (Fig. 3b). The editing effects of the simultaneous C-to-T and A-to-G point mutation at the same DNA strand were further analyzed at the M-mstn-G1, M-mstn-G2, and M-tyr-G3 targeting sites for MEFs and at the P-TYR-G1, P-TYR-G3, P-FANCA-G1, and P-LMNA-AC1 targeting sites for PFFs. For these targeting loci, the ACBE system performed higher efficiency of simultaneous C-to-T and A-to-G point mutation at the same DNA strand than the CBE + ABE system. The efficiencies of dual-function base editing at the same DNA strand ranged from 0.21 to 1.69% for ACBE and 0.0015 to 0.16% for CBE + ABE (Fig. 4c, d). These efficiencies were lower than those in HEK293 cells probably because of the low transfection frequency of primary somatic cells. Moreover, the top four POTs of M-mstn-G2, M-tyr-G3, P-TYR-G3, and P-LMNA-AC1 were selected to verify the off-target effects of ACBE by Sanger sequencing. The Sanger sequencing results showed that no detectable off-target effects were observed in all tested POTs (Additional file 1: Fig. S8). These results indicated that the ACBE system maintained the dual function of C-to-T and A-to-G point mutations and could induce simultaneous C-to-T and A-to-G substitution at the same DNA strand with high specificity in mouse and porcine primary somatic cells.

### Evaluation of base editing efficiency with different spacer lengths of sgRNA in the ACBE system

As previously reported, different spacer lengths of sgRNAs influenced the base editing effects of ABE [22]. Next, the effects of different sgRNA spacer lengths on the ACBE system were evaluated. Six sgRNAs with spacer lengths of 15, 18, 20, 24, 27, and 31 nt (referred to as S2-15, S2-18, S2-20, S2-24, S2-27, and S2-31) targeting the exogenous EGFP were designed and analyzed in the HEK293-EGFP cell line (Fig. 4a). The ACBE-expressing vectors and EGFP-Stop2 with different spacer lengths were co-transfected into the HEK293-EGFP cells. Five-day post transfection, the cells were collected for flow cytometry analysis. The results showed that the proportion of negative cells with S2-18 (40.27%) was similar to S2-20 (40.50%), whereas that of S2-15, S2-24, S2-27, and S2-31 were significantly reduced compared with S2-20 and S2-18 (Fig. 4b). Moreover, six sgRNAs with spacer lengths of 15, 18, 20, 24, 27, and 31 nt targeting the endogenous P53-G8 and P53-G9 loci (referred to as P53-G8-15, 18, 20, 24, 27, and 31 and P53-G9-15, 18, 20, 24, 27, and 31) were tested in HEK293 cells (Fig. 4c, d). Consistent with the results of EGFP, the sgRNAs of the P53-G8 and P53-G9 loci with 20 nt spacer length had optimal base editing effects in the editing windows (Fig. 4e, f). The sgRNAs with 15 nt spacer length could not support the base editing, similar to the reported effect of wild-type Cas9 [23]. The sgRNAs with longer spacer over 20 nt reduced the base editing efficiencies of C-to-T or A-to-G to a varying degree at the editing windows while slightly increased the base editing efficiencies at some bystander bases. In P53-G9 targeting locus, the sgRNA with 24 nt spacer length showed the highest efficiency of C-to-T point mutation at 1C (Fig. 4f). We also detected the effects of simultaneous C-to-T and A-to-G substitutions at the same DNA strand with different spacer lengths of sgRNAs by analyzing the corresponding read ratio of amplicon deep sequencing. The results showed that sgRNA with 20 nt spacer length had the optimal simultaneous C-to-T and A-to-G substitutions at the same DNA strand (0.006% of P53-G8-15, 0.09% of P53-G8-18, 2.36% of P53-G8-20, 1.58% of P53-G8-24, 1.23% of P53-G8-27, 1.99% of P53-G8-31, 0.017% of P53-G9-15, 0.67% of P53-G9-18, 2.41% of P53-G9-20, 0.095% of P53-G9-24, 0.70% of P53-G9-27, and 1.10% of P53-G9-31) (Fig. 4g, h). These results indicated that shortening or extending the sgRNA spacer length could significantly decrease the efficiency of simultaneous C-to-T or A-to-G point mutation at the same DNA strand.

### Different linker length influences the dual-function base editing effect of the ACBE system

**Fig. 5:**
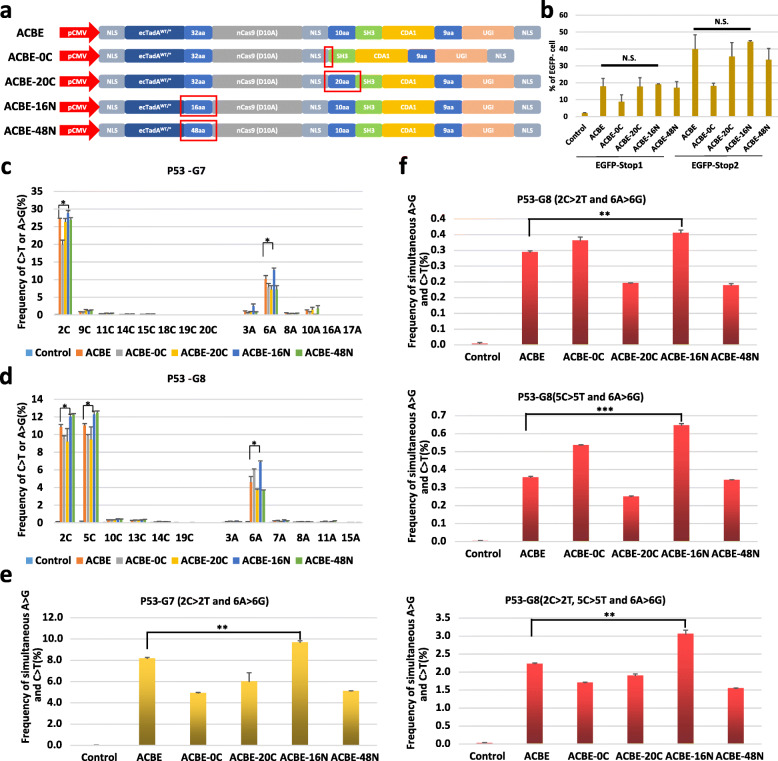
ACBE-mediated base editing effects with different lengths of the linker peptides. **a** Schematic representation of ACBE, ACBE-0C, ACBE-20C, ACBE-16N, and ACBE-48 N architecture. The red square frame indicated the change of linkers. **b** Summary of flow cytometry results of HEK293-EGFP cells transfected ACBE variants with EGFP-Stop1 and EGFP-Stop2. **c**, **d** Summary of ACBE variants-mediated base editing patterns and efficiencies of all C and A in P53-G7 (**c**) and P53-G8 (**d**) targeting sites. **e**, **f** Summary of ACBE-mediated simbase editing patterns and efficiencies of all C and A in P53-G7 (**e**) and P53-G8 (**f**) sites using sgRNAs with different spacer lengths. The abovementioned values and error bars indicate mean ± s.e.m. of three independent experiments. The controls were untreated cell samples

The linker peptide linked the components of base editors and maintained the balance of these functional domains. The length of the linker peptide might change the spatial position of ecTadA^WT/*^, nCas9, and PmCDA1 and then affect the efficiency of dual-function base editing. Next, the base editing effects of ACBEs with different lengths of the linker peptide between ecTadA^WT/*^ and nCas9 and between nCas9 and PmCDA1 were evaluated, respectively. As shown in Fig. 5a, three ACBE variants with 0, 10, and 20 amino acid (aa) linkers between nCas9 and PmCDA1 were designed and constructed (referred to as ACBE-0C, ACBE, and ACBE-20C). The linker peptide of these ACBEs between ecTadA^WT/*^ and nCas9 was all 32 aa length. Similarly, the linker peptide between nCas9 and PmCDA1 was identical to that of 10 aa length, whereas the length between ecTadA^WT/*^ and nCas9 was designed as 16, 32, and 48 aa (referred to as ACBE-16N, ACBE, and ACBE-48 N). The base editing efficiencies of these five ACBE variants were assessed at the exogenous EGFP and human endogenous *TP53* genes. In the HEK293-EGFP cell line, EGFP-Stop1 for C-to-T substitution and EGFP-Stop2 for A-to-G substitution were used. Flow cytometry analysis showed that all variants maintained the base editing ability of C-to-T and A-to-G substitution, whereas the ACBE-10C represented a significant reduction in the efficiency of C-to-T and A-to-G point mutations. Moreover, the average proportion of EGFP-negative cells in the ACBE-16N group was higher than that in the ACBE group with no significant differences (Fig. 5b). For endogenous *TP53* genes, P53-G7 and P53-G8 were tested. The amplicon deep sequencing results showed that different variants had different C-to-T and A-to-G point mutations. Notably, ACBE-16N showed more efficient base editing than ACBE in C-to-T and A-to-G substitutions in two targeting loci with a significant difference, whereas other variants showed a decreased efficiency in C-to-T and A-to-G point mutations or slightly increased efficiency in C-to-T or A-to-G mutation compared with ACBE (Fig. 5c, d). Furthermore, we evaluated whether ACBE variants could promote the simultaneous C-to-T and A-to-G point mutations at the same DNA strand. As shown in Fig. 5e, f, ACBE-16N could significantly increase the efficiency of heterogeneous mutation at the same DNA strand in P53-G7 (2C > 2T and 6A > 6G, 8.178% of ACBE and 9.696% of ACBE-16N) and P53-G8 (2C > 2T and 6A > 6G, 0.295% of ACBE and 0.356% of ACBE-16N; 5C > 5T and 6A > 6G, 0.357% of ACBE and 0.646% of ACBE-16N; and 2C > 2T, 5C > 5G, and 6A > 6G, 2.229% of ACBE and 3.061% of ACBE-16N) targeting loci. ACBE-0C could partially increase the efficiency of a simultaneous point mutation at the same DNA strand in two dual base editing patterns (2C > 2T and 6A > 6G, and 5C > 5T and 6A > 6G) of P53-G8, whereas ACBE-20C and ACBE-48 N did not show any improvement in simultaneous C-to-T and A-to-G point mutations at the same DNA strand (Fig. 5e, f). These results showed that changing the length of linkers could regulate the efficiency of C-to-T and A-to-G substitutions of the ACBE system, which provides a strategy to optimize the dual function of the ACBE system.

## Discussion

Previous reports showed that the dual-function base editors, STEMEs [16] and Target-ACE [17], could be used for simultaneous C-to-T and A-to-G base editing. In addition, during the revision process of this work, three independent studies reported that base editors fused with CBE and ABE (referred to as synchronous programmable adenine and cytosine editor (SPACE) [24], A&C-BEmax [25], Target-ACEmax [17] (previously posted in bioRxiv)). However, the abovementioned systems have only been evaluated in either plant cells or mammalian immortalized cells and remained to be tested in mammalian primary somatic cells.

In this study, using complementary structure features in a position of deaminases and editing windows between Target-AID and ABE7.10, we fused the two base editors and generated a new base editing tool, namely, the ACBE system. ACBE had the similar nucleotide position spectrum (+ 1 to + 7 bp) compared with Target-AID [11], whereas had narrowed editing spectrum (+ 4 to + 6 bp) compared with ABE7.10 [12]. The effectiveness test of simultaneous C-to-T and A-to-G substitutions in seven endogenous genes with the ACBE system showed that more than half (25/43) of sgRNAs, which covered both cytosine and adenine, could simultaneously achieve C-to-T and A-to-G conversions. The other 18 sgRNAs resulted in only C-to-T base editing, but no A-to-G conversion, indicating that the frequency of A-to-G conversion was lower than that of C-to-T conversion in the ACBE system, which was opposite to that when CBE + ABE was applied for base editing. PmCDA1 has a greater affinity of a single-strand DNA than TadA/TadA* [26, 27], meaning that it also could bind to nonspecific single-strand DNA more frequently, which has been used to explain why the CBE system would lead to much higher DNA random off-target than ABE system [28, 29]. Therefore, in CBE + ABE system, the higher DNA binding ability of PmCDA1 make some of PmCDA1 bind to the nonspecific single DNA strand randomly, which would reduce the number of PmCDA1 protein molecules to bind the desired target site, thus C-to-T conversion frequency is lower than that of A-to-G conversion in CBE + ABE system. However, in the ACBE system, PmCDA1 and TadA/TadA* were constructed in the same vector linked with nCas9, which made them have the same chance to present at the targeting site. Due to the higher DNA binding ability, PmCDA1 is easier to occupy the single DNA strand at the R-loop created by nCas9, resulting in a prior C-to-T to A-to-G in ACBE-mediated base editing. The unequal ability of DNA binding between the two deaminases reduced the overall efficiency of simultaneous C-to-T and A-to-G substitution in the ACBE system. Thus, the modification of PmCDA1 to reduce the DNA binding ability might be a potential approach to increase the efficiency of heterologous base editing at the same locus.

The somatic cell gene editing, followed by nuclear transfer, has become a routine approach to generate genetically modified animals. Our results showed that the ACBE system was also effective in MEFs and PFFs in creating dual conversions of C-to T and A-to-G. The overall gene editing effect was slightly lower than that of HEK293 cells, and this finding was reasonable because primary somatic cells had a limited ability to proliferate in vitro culture and a lower capacity to accept gene transfection compared with immortalized cell lines. The efficiency of the ACBE system for the simultaneous generation of C-to-T and A-to-G mutations in somatic cells was acceptable because it could produce sufficient cells for making genetically modified animals with heterogeneous base editing through a somatic cell nuclear transfer approach [30].

ACBEs with different lengths of linkers or sgRNAs had different base editing efficiencies in the exogenous and endogenous targeting loci. ACBE with 16 aa linker between ecTadA^WT^^/^^*^ and nCas9 had the optimal dual-functional base editing, possibly because 16 aa linker made the deaminases closer to targeting DNA, thereby increasing the base editing efficiency. In addition, similar to wild-type Cas9 and single-function base editors, 20 nt sgRNA had the highest simultaneous C-to-T and A-to-G base editing. Thus optimization of linkers and sgRNAs was a potential approach for increasing the efficiency of heterologous base editing at the same locus.

In this report, the ACBE system was successfully constructed by integrating two basic editors, which require NGG PAM. Some existing strategies could be used to expand the targeting scope of ACBE because previous studies have developed many variants of CRISPR-Cas protein requiring different PAM sequences or the restricted-less PAMs [31, 32]. Recently, C-to-G base editors consisted of a Cas9 nickase, a cytidine deaminase variant, and uracil DNA N-glycosylase (eUNG), which could induce targeted C-to-G transversions with a high editing specificity in mammalian cells [33, 34]. Similarly, new dual-function base editors could be generated to simultaneously induce C-to-G and A-to-G by using the strategies established in this study.

The off-target effect of a new gene-editing tool should be considered. As the components of the ACBE system were all derived from the existing single base editing system, the ACBE system might contain the off-target characteristics of the componential base editor, including the DNA and RNA off-target editing ability. In this study, we found that the proportion of indels and the DNA potential off-target effects in the ACBE system did not increase compared with that of the single base editor. The DNA random off-target and RNA off-target effects of the ACBE system were not detected in this study, which should be tested in future research.

## Conclusion

The ACBE system integrating the Target-AID and ABE7.10 into a single structure obtained the dual function of C-to-T and A-to-G substitutions and could efficiently induce heterogeneous base mutations at the same DNA strand with single sgRNA in immortalized and primary somatic cells. The application of the ACBE system would expand base editor toolkits and should promote the generation of gene editing animals and the gene therapy of genetic diseases with heterogeneous point mutations.

## Methods

### Vector construction

Plasmids expressing Target-AID (pcDNA3.1-Target-AID) and ABE7.10 (pcDNA3.1-ABE7.10) were obtained from our lab. The pcDNA3.1-ACBE expressing plasmid was constructed by cloning the PmCDA1 and UGI fragments amplified from pcDNA-Target-AID into the C terminus of plasmid pcDNA3.1-ABE7.10. The pcDNA3.1-ACBE-0C and pcDNA3.1-ACBE-20C were constructed similarly to pCDNA3.1-ACBE but with the 0 and 20 aa linkers, respectively. pcDNA3.1-ACBE-16N and pCDNA3.1-48 N were constructed by fusing the heterodimer of TadA with 16 and 48 aa linkers, respectively, into the N terminus of pcDNA3.1-Target-AID. These vectors were generated by using a recombination kit (ClonExpress® MultiS, Vazyme). All sgRNAs used in this study were designed in accordance with the G-N19-NGG rule. In brief, two complementary oligonucleotides of sgRNAs were synthesized and then annealed to double-stranded DNAs. The annealed products were then cloned into the BbsI-digested U6-sgRNA cloning vector, and the sgRNA-expressing plasmids were obtained. All newly constructed plasmids were confirmed by Sanger sequencing.

### Cell culture and transfection

HEK293 cells were cultured in Dulbecco’s modified Eagle’s medium (DMEM, HyClone) supplemented with 10% fetal bovine serum (FBS, Gibco), whereas the PFFs and MEFs were cultured in DMEM supplemented with 15% FBS, 1% nonessential amino acids (NEAA, Gibco), 2 mM GlutaMAX (Gibco), and 1 mM sodium pyruvate (Gibco). The culture dishes of HEK293 and MEFs were maintained at 37.5 °C with 5% CO_2_, and PFFs were maintained at 38.5 °C with 5% CO_2_.

Before electroporation, the cells were digested with 0.05% pancreatin (Gibco) and collected. Then, the collected cells were co-electroporated sgRNA-expressing vectors (4 μg) with pcDNA3.1-Target-AID (7.2 μg), pcDNA3.1-ABE7.10 (7.2 μg), pcDNA3.1-Target-AID+pcDNA3.1-ABE7.10 (7.2 μg+7.2 μg) or pCDNA3.1-ACBE (8 μg) at 1110 V, 30 ms, and 2 pulse (HEK293) or 1350 V, 30 ms, and 1 pulse (PFFs, MEFs) by using the Neon™ transfection system (Life Technology). The transfected cells were collected and sequenced 3 days later to detect the efficiency of base editing in genome. In addition, transfected HEK293-EGFP cells were detected with flow cytometry 5 days post-transfection by using BD Accuri C6.

### DNA extraction

Whole DNAs were extracted from the cells by using two approaches. (1) Few cells were collected and lysed in 10 μL of lysis buffer (0.45% NP-40 plus 0.6% proteinase K) at 56 °C for 60 min and then at 95 °C for 10 min. (2) For a large amount of cells, genomic DNAs were extracted by using a TIANamp Genomic DNA kit (TIANGEN) in accordance with the manufacturer’s instructions. The lysate or extracted DNA was then used as a PCR template.

### TA clone sequencing

The target sequence was first generated from the genome of transfected cells by PCR with the 2 × Phanta Max Master mix (Vazyme) and specific primer. Then, an adenine deoxyribonucleotide was added to the PCR products with Taq polymerase at the 72 °C for 10 min (10 μL reaction mix, consisting of 5 μL 2 × Rapid Taq Master Mix (Vazyme) and 5 μL PCR product). The final PCR product was ligased to pMD18-T Vector (TaKaRa) with Solution I (TaKaRa) at 16 °C for 1 h (10 μL reaction mix, consisting of 4.5 μL final PCR product, 0.5 μL pMD18-T Vector and 5 μL Solution I). The ligation product was transfected to 50 μL DH5α cells and cultured at 37 °C until the proper single bacteria colony occurred. Ten single bacterial colonies were sent for vector extraction and then Sanger sequencing.

### Amplicon deep sequencing and data analysis

The detecting target region was initially amplified through PCR from the extracted DNA of the transfected cells with the corresponding site-specific primers. Each PCR was performed in 20 μL volume comprising 1 μL of the template, 1 μL of 10 μM each primer, and 10 μL 2 × Phanta master mix with the following thermal cycler conditions: 95 °C for 3 min; 12 cycles of 95 °C for 10 s, 55 °C for 10 s, and 72 °C for 10 s; followed by 72 °C for 5 min as a final extension; and 12 °C for hold. The products of the first reaction were used as the PCR template, and the second PCR amplification was performed in 40 μL volume containing 2 μL of the template and the custom Illumina index primers with the following thermal cycler conditions: 95 °C for 3 min; 30 cycles of 95 °C for 10 s, 55 °C for 10 s, and 72 °C for 10 s; 72 °C for 5 min as a final extension; and 12 °C for hold. All the amplified products in different target regions were electrophoresed in 1.5% agarose gels with 1 × TAE buffer, and the target bands were cut for extraction by using a HiPure Gel Pure DNA Mini kit (Magen). The concentration of purified products was quantified with an Ipure Qubit dsDNA HS assay kit (IGE Biotech). Then, equivalent amounts of the purified products were mixed to produce an Illumina sequencing library. The library was sent to IGE Biotech (Guangzhou) for amplicon deep sequencing by using an Illumina HiSeq 2500 platform. The protospacer sequences in the reads were investigated to identify C-to-T, A-to-G, simultaneous C-to-T and A-to-G point mutations, and indels. The presented ratio was calculated by comparing the individual reads to the whole reads. The amplicons were sequenced in three of the repeated assays for each target site. Amplicon reads with a quality score of < 30 were filtered.

### Off-target analysis

The POTs of each sgRNA were predicted to analyze site-specific edits by the base editing systems via an online design tool (http://www.rgenome.net/cas-offinder/) [35] allowing for un-gapped alignment with up to three mismatches in the sgRNA target sequence. All POTs were amplified via PCR and then subjected to Sanger sequencing to confirm the off-target effects.

### Statistical analysis

Data were statistically analyzed by using GraphPad Prism v.7.0 and Excel. The average editing frequencies of different cytosines and adenines in the editing window were analyzed on the online tool, EditR 1.0.9 (https://moriaritylab.shinyapps.io/editr_v10/) [21]. The numerical values of target sites were presented as means ± s.e.m. or means.

## Supplementary information

**Additional file 1: Figure S1-S8. Figure S1**-[Detecting the expression of base editors]. **Figure S2**-[The ACBE system mediated heterologous C-to-T plus A-to-G substitutions in HEK293-EGFP cells]. **Figure S3**-[The preference analysis of ACBE]. **Figure S4**-[Comparation of different base editor-mediated base editing patterns and efficiencies of all C and A in P53-G7, P53-G8, LMNA-G1, LDHA-G1 and PGK1-G1 targeting sites]. **Figure S5**-[Summary of mutation patterns and efficiencies of cells transfected CBE or ABE with P53-G7, P53-G8, LMNA-G1, LDHA-G1 and PGK1-G4, respectively]. **Figure S6**-[Off-target analysis of different base editing system]. **Figure S7** [Off-target analysis of ACBE system]. **Figure S8**-[Off-target analysis of ACBE system in MEFs and PFFs].

**Additional file 2 : Tables S1-S2. TableS1**-[sgRNAs used in this study]. **TableS2**-[The nucleotides in positions 4–6 of 18 sgRNAs with only C-to-T conversions mediated by the ACBE system].

## Data Availability

The high-throughput sequencing data are available at the Sequence Read Archive (PRJNA601637) of the NCBI [36]. The authors declare that all data supporting the findings of this study are available in the article and its supplementary figures and tables. Such data are also available from the corresponding author upon request.
