# Supplementary material for: ACBE, a new base editor for simultaneous C-to-T and A-to-G substitutions in mammalian systems
Source: BMC Biol. 2020 Sep 23;18:131. doi: 10.1186/s12915-020-00866-5 (PMC7510086; doi:10.1186/s12915-020-00866-5)
Supplement: Supplementary file 1 — Additional file 1: Figure S1-S8. Figure S1-[Detecting the expression of base editors]. Figure S2-[The ACBE system mediated heterologous C-to-T plus A-to-G substitutions in HEK293-EGFP cells]. Figure S3-[The preference analysis of ACBE]. Figure S4-[Comparation of different base editor-mediated base editing patterns and efficiencies of all C and A in P53-G7, P53-G8, LMNA-G1, LDHA-G1 and PGK1-G1 targeting sites]. Figure S5-[Summary of mutation patterns and efficiencies of cells transfected CBE or ABE with P53-G7, P53-G8, LMNA-G1, LDHA-G1 and PGK1-G4, respectively]. Figure S6-[Off-target analysis of different base editing system]. Figure S7 [Off-target analysis of ACBE system]. Figure S8-[Off-target analysis of ACBE system in MEFs and PFFs]. [file 12915_2020_866_MOESM1_ESM.pdf]

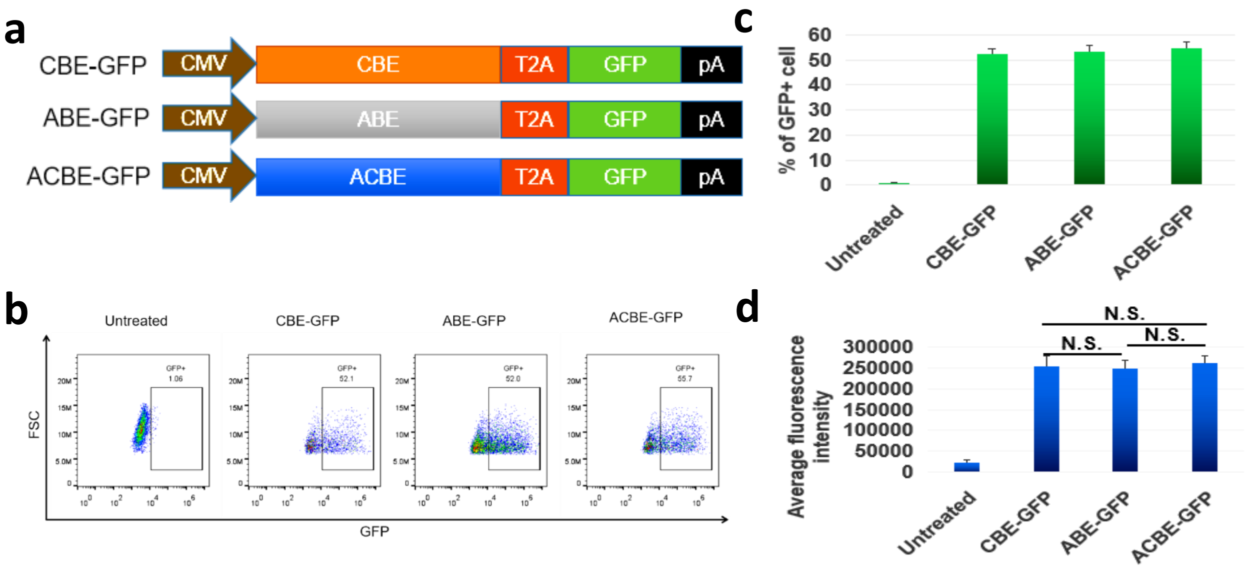

Figure. S1 Detecting the expression of base editors. (**a**) Schematic structure of base editor fusing to GFP with the T2A. The expression of GFP indicated the expression of the fused base editor. (**b**) Frequency of GFP^+ cells transfected with plasmid CBE-GFP, ABE-GFP and ACBE-GFP, respectively were quantified by flow cytometry. Untreated cell sample served as the negative control. (**c**) Summary of the GFP^+ frequencies in (c) of three independent repeat experiments. The values and error bars showed the mean ± s.e.m. (**d**) Average fluorescence intensity of GFP+ cells transfected with plasmid CBE-GFP, ABE-GFP and ACBE-GFP indicated the expression intensity of corresponding base editor. N.S., not significant(P>0.05, unpaired two tailed t-test).

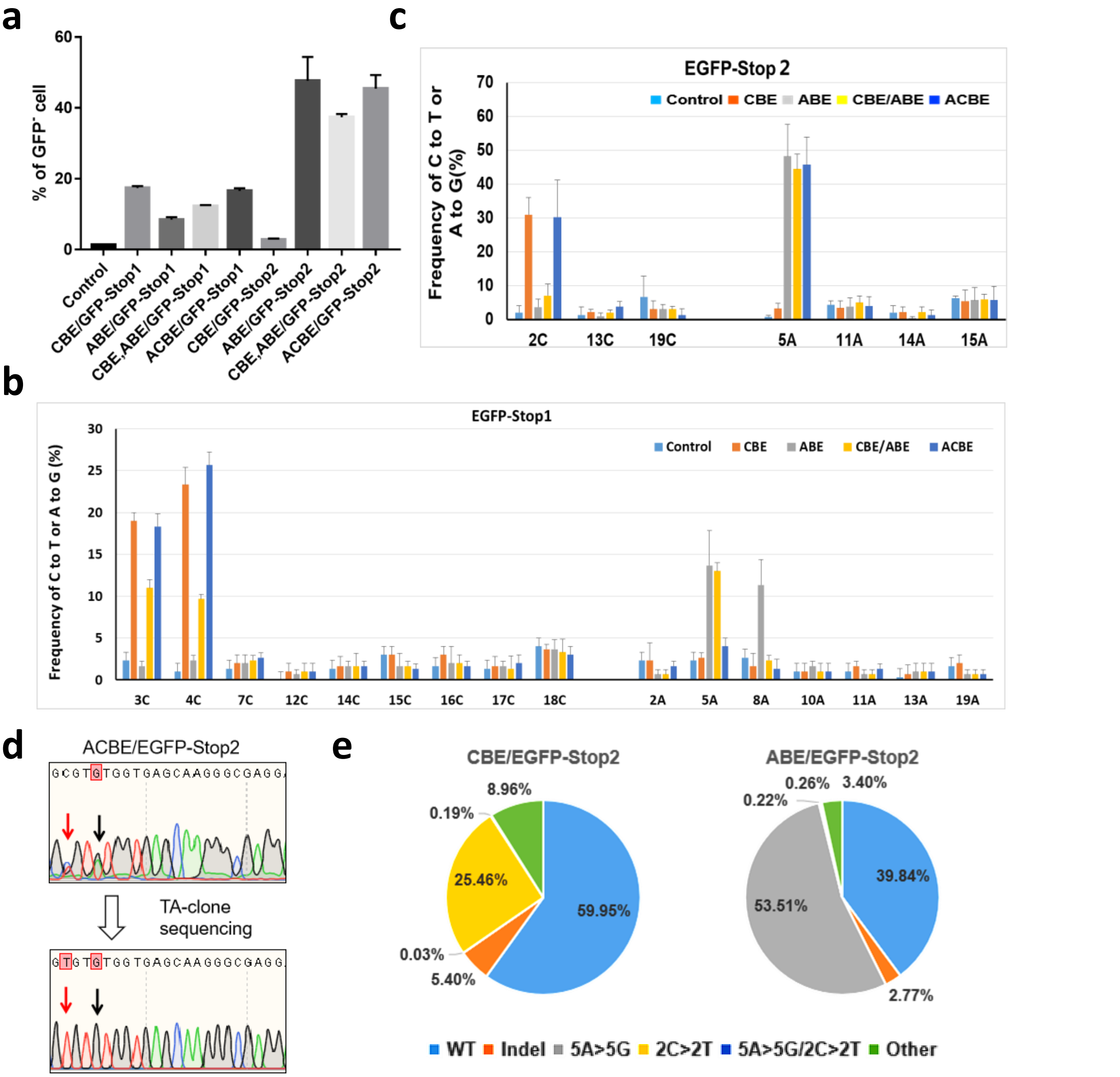

Figure S2. The ACBE system mediated heterologous C-to-T plus A-to-G substitutions in HEK293-EGFP cells. (**a**) Summary of flow cytometry results of HEK293-EGFP cells transfected CBE, ABE, CBE+ABE, and ACBE with EGFP-Stop1 and EGFP-Stop2, respectively. Values and error bars indicate mean ± s.e.m. of three independent experiments. An untreated cell sample served as control. (**b, c**) Summary of base editing patterns and efficiencies of all C and A in EGFP-Stop1 (**b**) and EGFP-Stop2 (**c**) targeting sites(n=3).Values and error bars indicate mean ± s.e.m. of three independent experiments. Control was un-transfected cell sample (**d**) Represented sanger sequencing results of TA-cloning for ACBE. (**e**) Summary of mutation patterns and efficiencies of cells transfected CBE or ABE with EGFP-Stop2. The result was based on the representive amplicon sequencing(n=2).

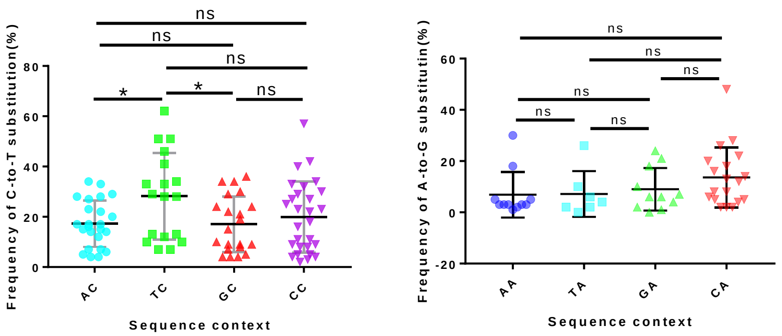

Figure S3. The preference analysis of ACBE.

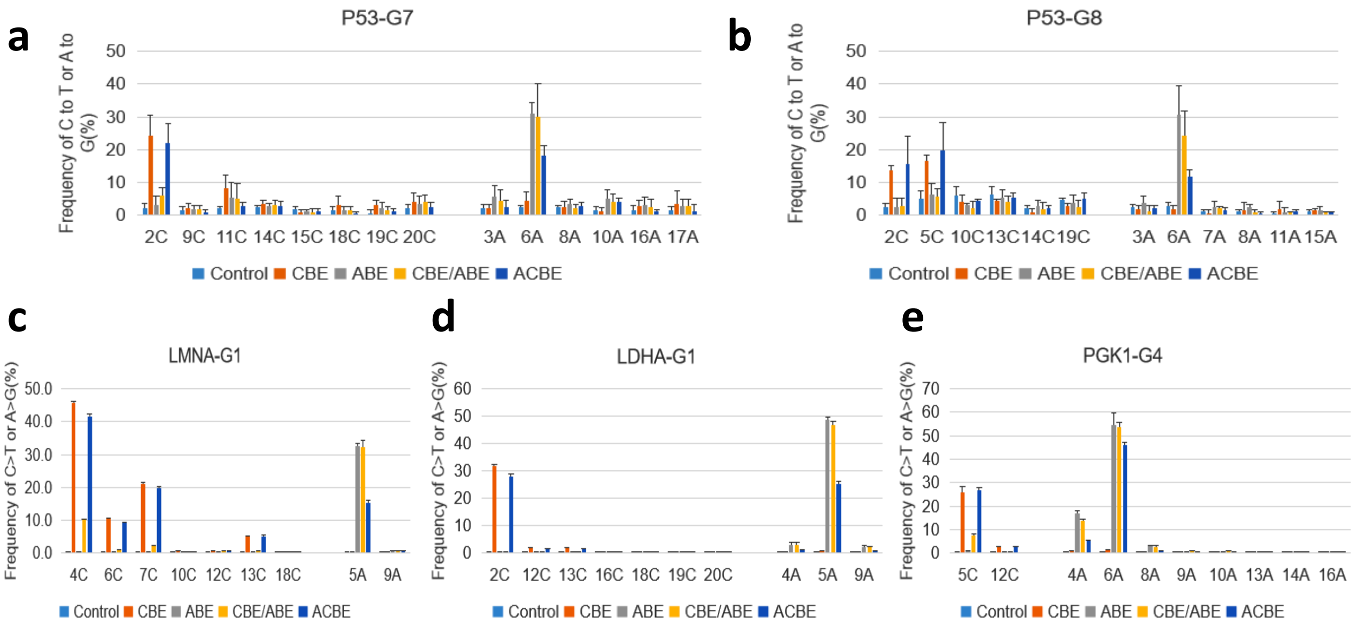

Figure S4. Comparation of different base editor-mediated base editing patterns and efficiencies of all C and A in *P53*-G7 (a), *P53*-G8 (b), *LMNA*-G1 (c), *LDHA*-G1 (d) and *PGK1*-G1 (e) targeting sites. (**a, b**) The base editing frequency was quantified from the results of Sanger sequencing with EditR. (**c, d, e**) The base editing frequency was quantified from the results of amplicon sequencing. The values and error bars above indicate mean ± s.e.m. of three independent experiments. An untreated cell sample served as control for the loci.

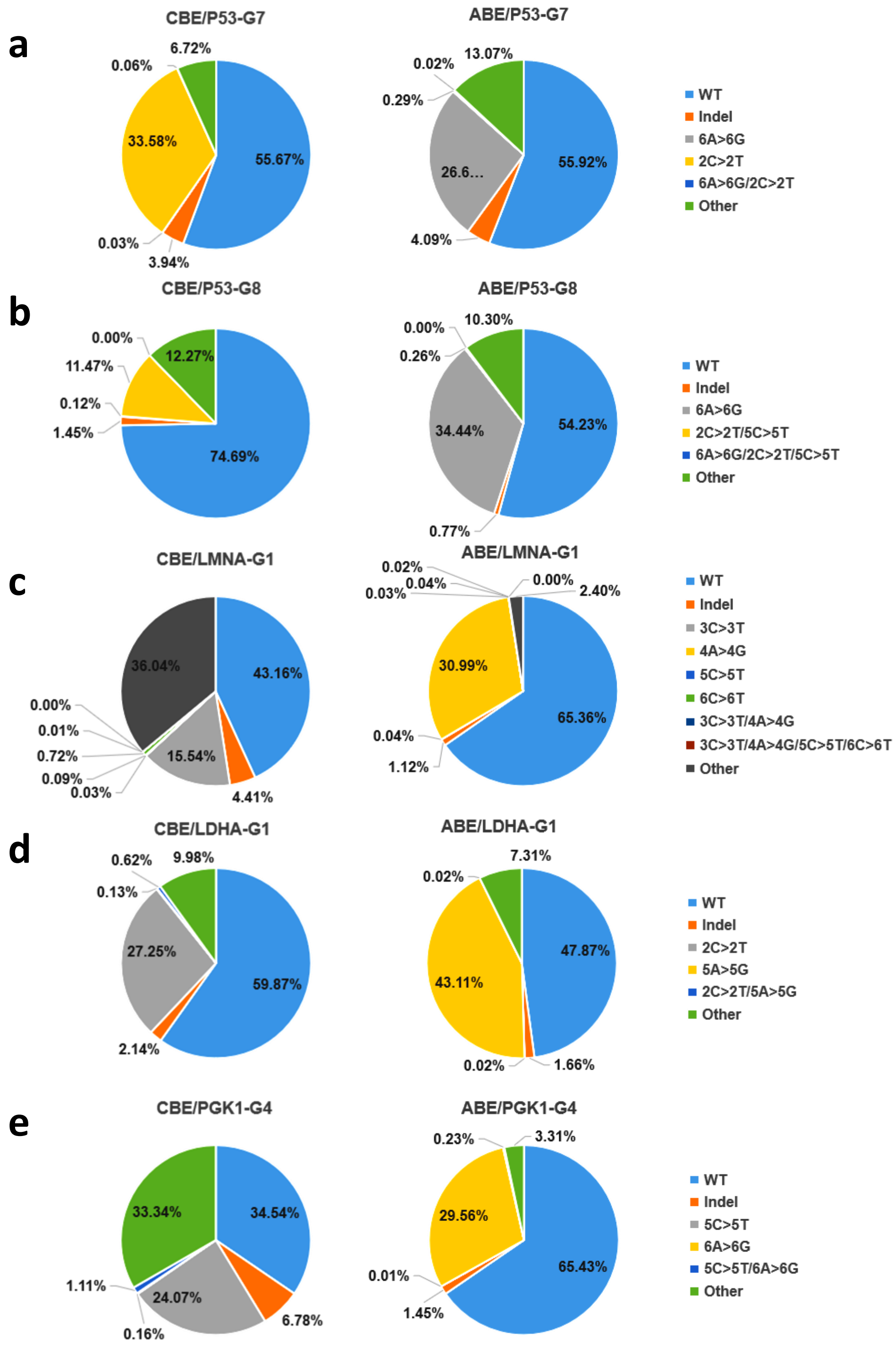

Figure S5. Summary of mutation patterns and efficiencies of cells transfected CBE or ABE with *P53*-G7 (a), *P53*-G8 (b), *LMNA*-G1 (c), *LDHA*-G1 (d) and *PGK1*-G4 (e), respectively. The ratio of different mutation patterns were calcualted basing on the reads from NGS of amplicons.

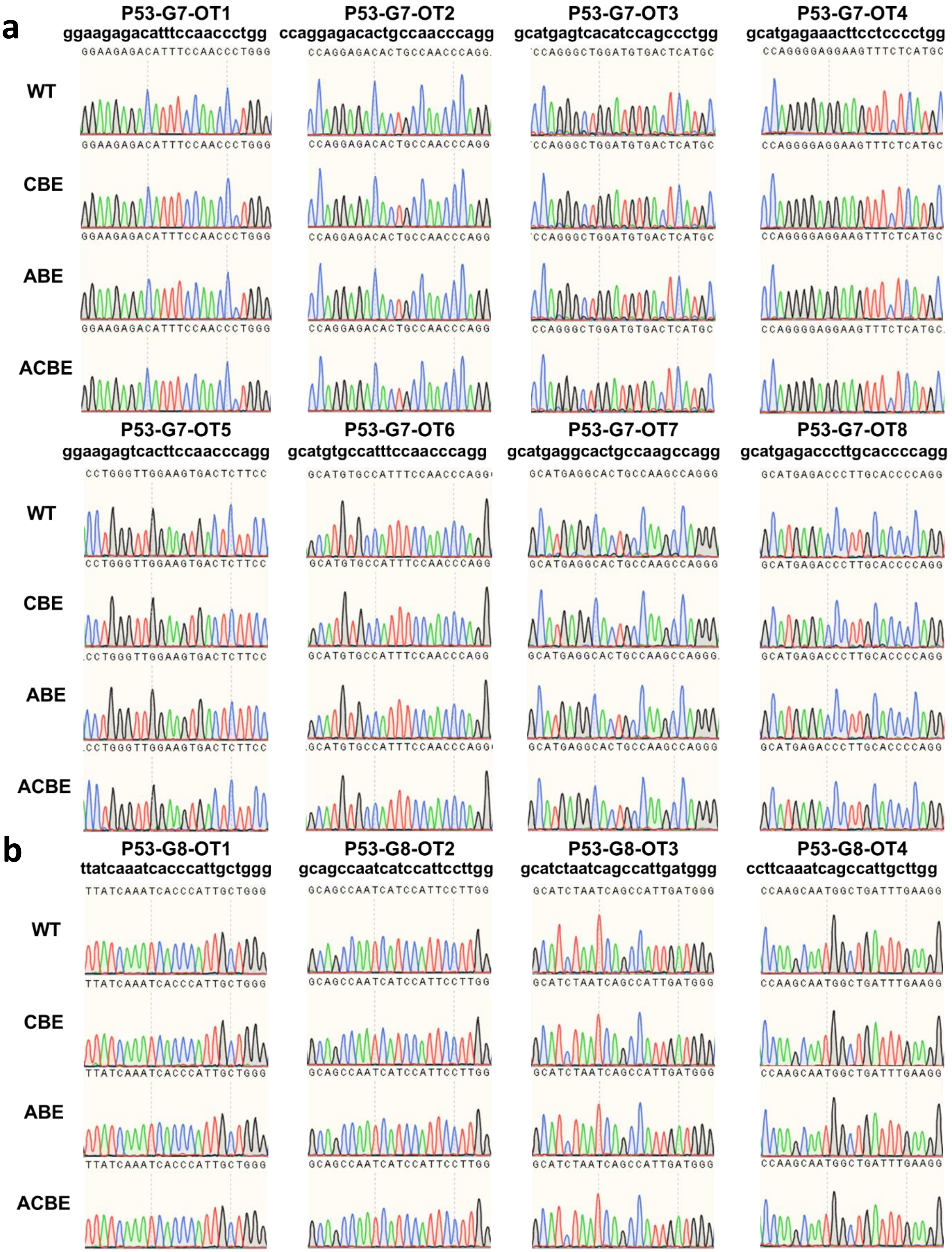

Figure S6. Off-target analysis of different base editing system. (**a**) Sanger sequencing results of 8 potential off-target sites of *P53*-G7. (**b**) Sanger sequencing results of 4 potential off-target sites of *P53*-G8.

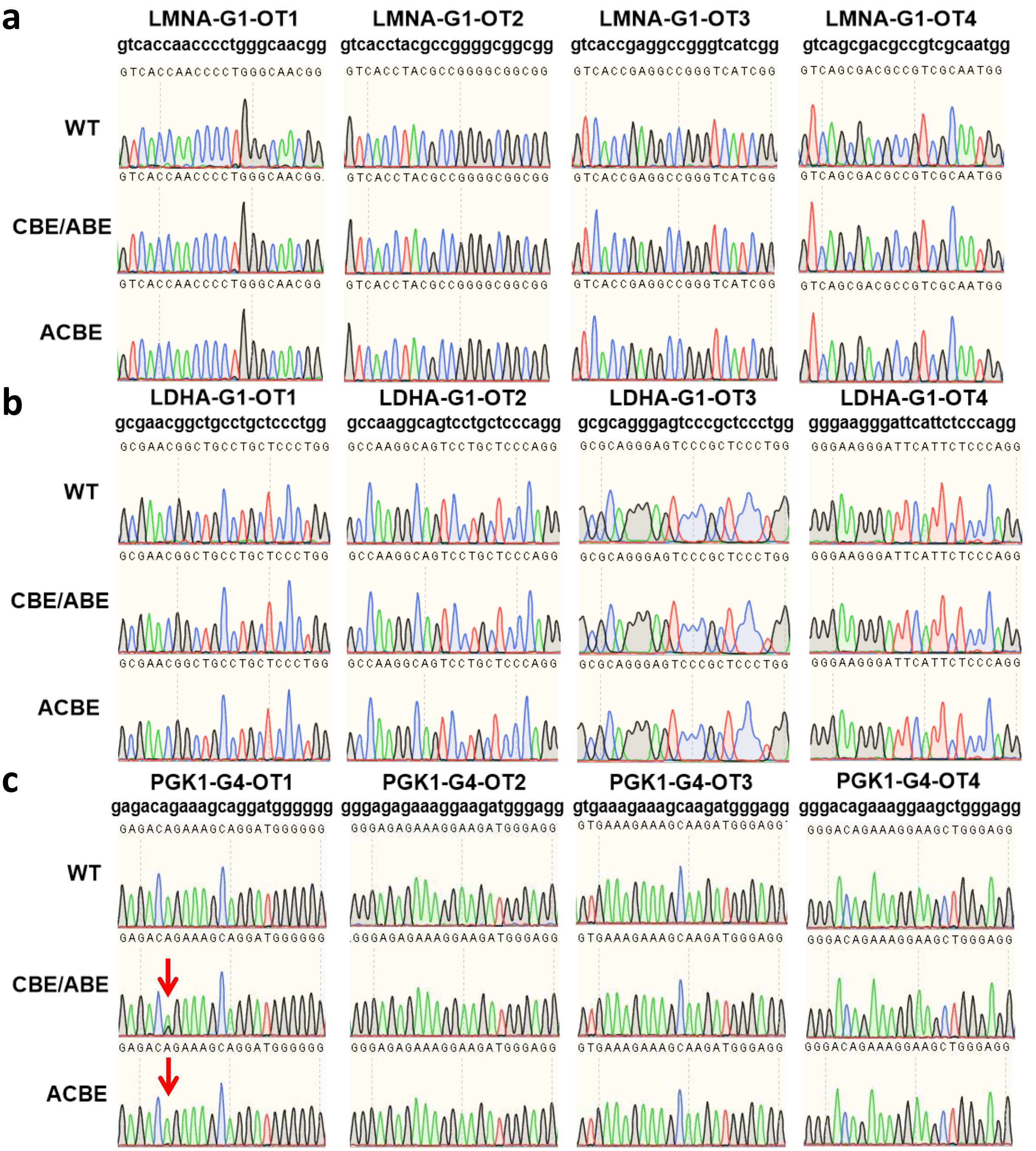

Figure S7. Off-target analysis of ACBE system. (**a, b, c**) Sanger sequencing results of 4 potential off-target sites each for *LMNA*-G1, *LDHA*-G1 and *PGK1*-G4. Red arrow indicated the off-target effect.

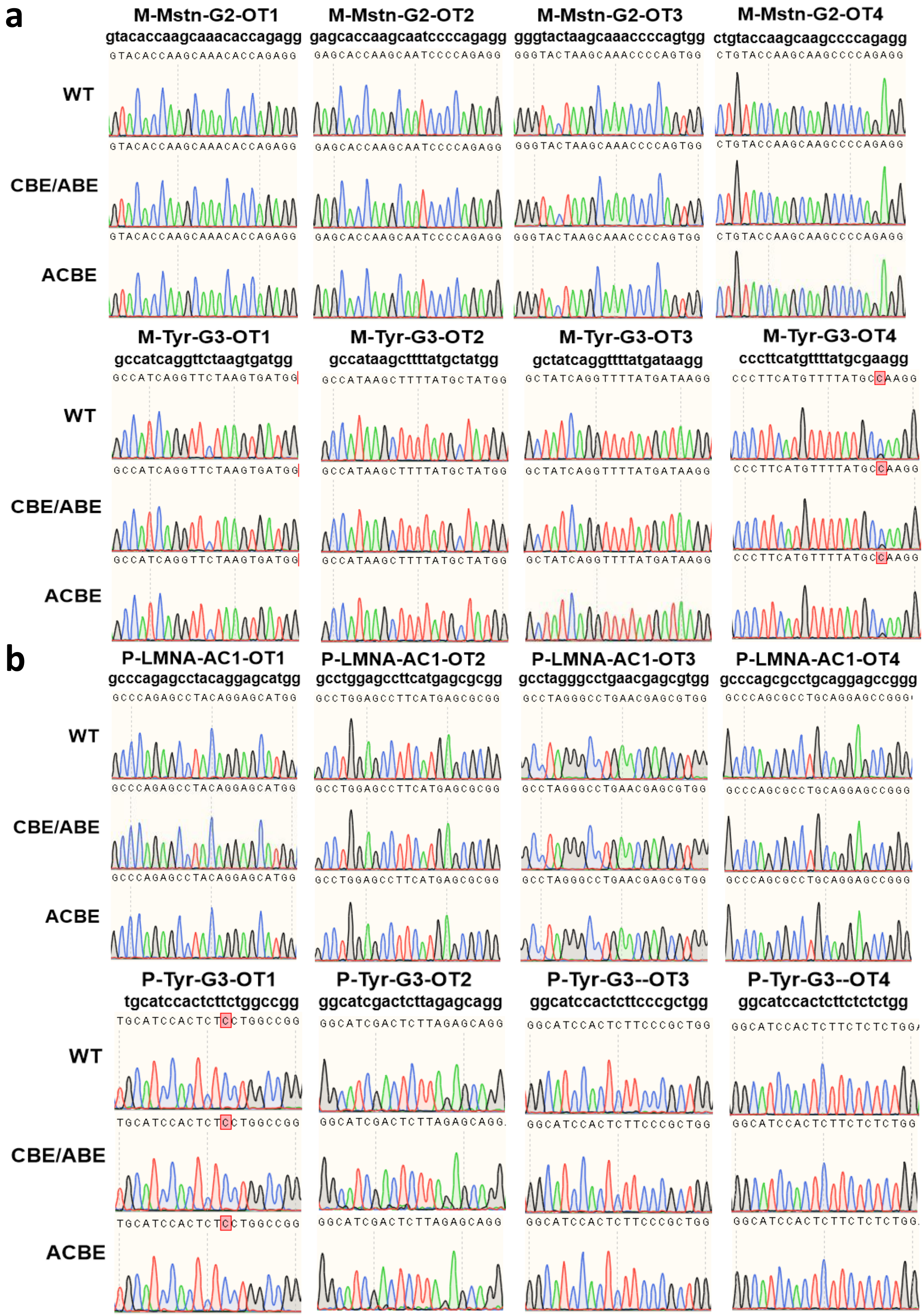

Figure S8. Off-target analysis of ACBE system in MEFs and PFFs (**a**) Sanger sequencing results of 4 potential off-target sites each for M-*Mstn*-G2 and M-*Tyr*-G3. (**b**) Sanger sequencing results of 4 potential off-target sites each for P-*LMNA*-AC1 and P-*Tyr*-G3. Different peak represents different base.
