# Supplementary material for: ACBE, a new base editor for simultaneous C-to-T and A-to-G substitutions in mammalian systems
Source: BMC Biol. 2020 Sep 23;18:131. doi: 10.1186/s12915-020-00866-5 (PMC7510086; doi:10.1186/s12915-020-00866-5)
Supplement: Supplementary file 2 — Additional file 2 : Tables S1-S2. TableS1-[sgRNAs used in this study]. TableS2-[The nucleotides in positions 4–6 of 18 sgRNAs with only C-to-T conversions mediated by the ACBE system]. [file 12915_2020_866_MOESM2_ESM.pdf]

	Name of sgRNA	Sequence		Name of sgRNA	Sequence
1	P53-G1	gcaagaaaaagaaataatgaGGG	29	PGK1-G7	ccacctgttgaatgaaagccAGG
2	P53-G2	gctaggaaagaggcaaggaaAGG	30	PGK1-G8	accttccaggagctccaaacTGG
3	P53-G3	gacagaagatgacaggggccAGG	31	PGK1-G9	catacccgcccaggatggccAGG
4	P53-G4	gatcaaaataaaggtggggtCGG	32	PGK1-G10	gactcaccatccagccagcAGG
5	P53-G5	ggacaaaggtccggaagttgTGG	33	PGK1-G11	gctaagcagattgtgtggaaTGG
6	P53-G6	ggtcagaggcaagcagaggcTGG	34	PGK1-G12	gtagaaatattttagtgataAGG
7	P53-G7	gcatgagacacttccaacccTGG	35	PGK1-G13	gtctacaaaaaaaaattagcTGG
8	P53-G8	gcatcaaatcatccattgctTGG	36	PGK1-G14	gatgattattggtggtggaaTGG
9	P53-G9	ctccatggcagtgacccggaAGG	37	LMNA-G1	gtcaccgacgccggggcaaAGG
10	P53-G10	atccattgcttgggacggcaAGG	38	LMNA-G2	gaacactgagtcaccgacgccGGG
11	P53-G11	aaacatcttgttgagggcagGGG	39	LMNA-G3	ggtctccatggccggcaggtTGG
12	P53-G12	gatggccatggcgcggacgcGGG	40	LMNA-G4	cacctgggctcctgagccgcTGG
13	P53-G13	gccgccggtgtaggagctgcTGG	41	AAVS1-G1	gctggcatccagactggctcTGG
14	P53-G14	gcttcccacaggtctctgctAGG	42	AAVS1-G2	gctgcaccccagctctaaggAGG
15	P53-G15	gccccctagcagagacctgtGGG	43	CTNNB1-G1	attacaggtggtggttaataAGG
16	LDHA-G1	gcgaagggattcctgctcccGGG	44	CTNNB1-G2	cttacccaagcattttcaccAGG
17	LDHA-G2	gcagaagaatcgcttaaaccTGG	45	CTNNB1-G3	ttaccagcttctacaatagcCGG
18	LDHA-G3	gctgatttataatcttctaaAGG	46	M-Mstn-G1	gatcagtacgacgtccagaGGG
19	LDHA-G4	taccttcattaagatactgaTGG	47	M-Mstn-G2	gtgcaccaagcaaaccccagAGG
20	TDP43-G1	gctgaaattaggtatttcctTGG	48	M-Mstn-G4	gataatccagtcccatccaaAGG
21	TDP43-G2	gtcaagaaagatcttaagacTGG	49	M-TYR-G1	gacctcagttccccttcaaaGGG
22	TDP43-G3	gtactgaatcacagctttgtAGG	50	M-TYR-G3	gccatcaggttttatgcgaTGG
23	PGK1-G1	gtcaatgagatgattattggTGG	51	P-Tyr-G1	gtggccagctctcaggcagGGG
24	PGK1-G2	ggccagatagttaatattttAGG	52	P-Tyr-G3	ggcatcgactcttcttgcTGG
25	PGK1-G3	ggaacaaaaatggggaacaaTGG	53	P-FANCA-G1	gctttgcaggatcaagcctcgCGG
26	PGK1-G4	gggacagaaagcaagatgggAGG	54	P-LMNA-AC1	gcctagagcctgcaggagcgCGG
27	PGK1-G5	gatactaaagaccagaatagTGG	55	P-LMNA-RE	cacctgggctcccgagctgcTGG
28	PGK1-G6	acaccgcgcgggcaggaacaGGG			

Table S1. sgRNAs used in this study.

sgRNA	1	2	3	4	5	6	7	8	9	10	11	12	13	14	15	16	17	18	19	20
P53-G2	G	C	T	A	G	G	A	A	A	G	A	G	G	C	A	A	G	G	A	A
P53-G13	G	C	C	G	C	C	G	G	T	G	T	A	G	G	A	G	C	T	G	C
P53-G14	G	C	T	T	C	C	C	A	C	A	G	G	T	C	T	C	T	G	C	T
P53-G15	G	C	C	C	C	T	A	G	C	A	G	A	G	A	C	C	T	G	T	
LDHA-G4	T	A	C	C	T	T	C	A	T	T	A	A	G	A	T	A	C	T	G	A
TDP43-G1	G	C	T	G	A	A	A	T	T	A	G	G	T	A	T	T	T	C	C	T
TDP43-G3	G	T	A	C	T	G	A	A	T	C	A	C	A	G	C	T	T	T	G	T
PGK1-G2	G	G	C	C	A	G	A	T	A	G	T	T	A	A	T	A	T	T	T	T
PGK1-G6	A	C	A	C	C	G	C	G	C	G	G	G	C	A	G	G	A	A	C	A
PGK1-G7	C	C	A	C	C	T	G	T	T	G	A	A	T	G	A	A	A	G	C	C
PGK1-G8	A	C	C	T	T	C	C	A	G	G	A	G	C	T	C	C	A	A	A	C
LDHA-G3	G	C	T	G	A	T	T	T	A	T	A	A	T	C	T	T	C	T	A	A
PGK1-G13	G	T	C	T	A	C	A	A	A	A	A	A	A	A	A	T	T	A	G	C
LMNA-G3	G	G	T	C	T	C	C	A	T	G	G	C	C	G	G	C	A	T	T	G
LMNA-G4	C	A	C	C	T	G	G	G	C	T	C	C	T	G	A	G	C	C	T	C
AAVS1-G1	G	C	T	G	G	C	A	T	C	C	A	G	A	C	T	G	G	C	T	C
CTNNB1-G2	C	T	T	A	C	C	C	A	A	G	C	A	T	T	T	T	C	A	C	C
CTNNB1-G3	T	T	A	C	C	A	G	C	T	T	C	T	A	C	A	A	T	A	G	C

Table S2. The nucleotides in positions 4-6 of 18 sgRNAs with only C-to-T conversions mediated by the ACBE system.
